# Clinical response to 2 protocols of aerosolized gentamicin in 46 dogs with *Bordetella bronchiseptica* infection (2012‐2018)

**DOI:** 10.1111/jvim.15843

**Published:** 2020-08-13

**Authors:** Aude Morgane Canonne, Elodie Roels, Maud Menard, Loïc Desquilbet, Frédéric Billen, Cécile Clercx

**Affiliations:** ^1^ Department of Clinical Sciences, Faculty of Veterinary Medicine University of Liège Liège Belgium; ^2^ Small Animals Internal Medicine Unit National Veterinary School of Alfort Maisons‐Alfort France; ^3^ Unit of Biostatistics National Veterinary School of Alfort Maisons‐Alfort France

**Keywords:** aerosol, *Bordetella bronchiseptica*, bronchoalveolar lavage, canine infectious respiratory disease (CIRD), dog, gentamicin, nebulization

## Abstract

**Background:**

*Bordetella bronchiseptica* (*Bb*) infection commonly causes respiratory disease in dogs. Gentamicin delivered by aerosol maximizes local drug delivery without systemic absorption but clinical response to protocols remains undetermined.

**Objectives:**

To compare the clinical response to 2 protocols of aerosolized delivery of gentamicin in bordetellosis.

**Animals:**

Forty‐six dogs with *Bb* infection confirmed by culture or quantitative polymerase chain reaction on bronchoalveolar lavage.

**Methods:**

Retrospective study. Administration of aerosolized gentamicin for ≥10 minutes q12h for ≥3 weeks using 4 mg/kg diluted with saline (group 1) or undiluted 5% solution (group 2). Clinical response firstly assessed after 3‐4 weeks and treatment pursued by 3‐weeks increments if cure not reached. Cure defined as absence of cough persisting at least a week after treatment interruption.

**Results:**

Demographic data were similar between both groups. Clinical cure at 3‐4 weeks was more frequently observed with the use of undiluted solution (19/33 vs 3/13 dogs, *P* = .03) in association with a shorter median duration of treatment (4 vs 6 weeks, *P* = .01). Dogs from group 2 having less than 1000 cells/μL in lavage were also more likely to be cured at 3‐4 weeks than dogs with more than 1000 cells/μL [9/9 vs 10/19, *P* = .006] and median duration of treatment in that subgroup of animals was reduced (3 vs 5 weeks, *P* = .02).

**Conclusion and Clinical Importance:**

Aerosolized delivery of gentamicin seems effective for inducing clinical cure in *Bb* infection. Clinical response appears better using undiluted 5% solution, particularly in the subgroup of dogs having less than 1000 cells/μL in lavage.

**Abbreviations**
BALF: bronchoalveolar lavage fluid
Bb: *Bordetella bronchiseptica*
CIRD‐C: canine infectious respiratory disease complex
*M*. *cynos*: *Mycoplasma cynos*
qPCR: quantitative PCR

## 1 INTRODUCTION

Despite the widespread availability of vaccines, *Bordetella bronchiseptica* (*Bb*) is still commonly documented as one of the primary causative agents of canine infectious respiratory disease complex (CIRD‐C) affecting mainly young dogs, especially in boarding kennels.^1^, ^2^, ^3^, ^4^, ^5^, ^6^, ^7^, ^8^ This contagious disease can be often self‐limiting, but a wide range of respiratory signs have been described, from mild illness to severe pneumonia and potential death.^8^, ^9^, ^10^, ^11^ As both symptomatic and carrier dogs can be a source of infection for other dogs and immune‐compromised owners,^12^, ^13^, ^14^ early diagnosis and prompt efficient treatment are required.

The clinical severity of *Bb* infection depends on several factors including bacterial load, strain virulence, viral or bacterial coinfection, immune status and airways comorbidities including laryngeal or tracheal anbormalities.^1^, ^2^, ^3^, ^4^ Implication of *Mycoplasma cynos* (*M*. *cynos*) in CIRD‐C is increasingly being recognized^5^, ^7^, ^15^, ^16^, ^17^, ^18^ but it is possible exacerbating role in dogs with *Bb* infection is unclear. Until now, the impact of clinical or other factors on therapeutic response was unknown in dogs with *Bb* infection. Among others, young age, bacterial coinfection, previous steroid treatment could rationally be suspected to negatively influence the clinical improvement.

While acute illness is the most common presentation, chronic and refractory *Bb* infection can also develop in dogs as a result of different mechanisms including adherence of *Bb* to cilia, induction of ciliostasis, biofilms and local immunosuppression.^19^, ^20^, ^21^, ^22^, ^23^, ^24^ Therapeutic concentrations of antimicrobial drugs could not be reached on the apical surface of bronchial epithelium, even if isolates are known to be *in vitro* susceptible to systemic drugs such as doxycycline or enrofloxacin.^25^ In humans, aerosolized delivery of aminoglycosides is now considered as one of cornerstones for treatment of bacterial infections in patients with various bronchopneumopathies^26^, ^27^, ^28^ with nebulizers producing adequate size of droplets able to reach lower airways.^29^, ^30^ Although nebulization of gentamicin is largely used in practice for respiratory infections in dogs, a single study about aerosolized delivery of gentamicin for *Bb* infection in dogs has been published 3 decades ago.^31^ Bacterial load decreased immediately after first administrations but increased within days of stopping treatment; protocols also varied between dogs with treatment and follow‐up periods being unfortunately short.^31^

The first objective of our study was to describe the clinical response to 2 protocols of gentamicin nebulizations in dogs with *Bb* infection that showed only poor improvement with previous oral antimicrobial treatment. Secondly, possible factors that could be associated with clinical response to aerosolized delivery of gentamicin were evaluated including age, breed, acute presentation (cough for less than 2 weeks), presence of alveolar radiographic lesions, previous steroid treatment, bacterial co‐infections, total cellularity and severity of neutrophilic inflammation of the lavage, concomitant oral antimicrobial treatment, use of cage vs facial mask, concomitant abnormal airways conformation and type of protocol.

## 2 MATERIALS AND METHODS

### 2.1 Case selection and data collection

Client‐owned dogs presented to Veterinary Small Animal Teaching Hospitals of the University of Liege (between January 2012 and December 2018) and the National School of Alfort (between October 2016 and December 2018), diagnosed with *Bb* infection, either by positive culture or quantitative polymerase chain reaction (qPCR) on bronchoalveolar lavage fluid (BALF) were included. All recruited dogs had received previous antimicrobial treatment. Data were retrospectively collected from the medical records and included signalment, body weight, type and date of previous vaccination against *Bb* if available, previous oral antimicrobial treatment and response, ongoing oral antimicrobial treatment, any previous steroid treatment, duration and severity of clinical signs, physical examination findings, results of blood CBC, radiographic lesions and findings from bronchoscopy and BALF analysis. Dogs were excluded if owner compliance was deemed suboptimal regarding the gentamicin nebulization protocol or if clinical follow up was unavailable.

Bronchoscopy, bronchoalveolar lavage procedure and BALF laboratory processing were performed as described earlier.^8^, ^32^ The recovered BALF was immediately processed. Aliquots of naïve BALF were used for conventional quantitative culture, for *Bb* and *M*. *cynos* qPCR, for total cell count (TCC) determination using a hemocytometer, as well as for cytospin preparation (centrifugation at 1400 rpm, 197*g*, for 4 minutes at 20°C, Thermo Shandon Cytospin 4) that was thereafter stained with a May‐Grünwald‐Giemsa stain. Normal TCC was considered to be 400‐600/μL; neutrophilic inflammation was concluded if >12% of neutrophils were observed. Samples with cytological evidence of oropharyngeal contamination were not interpreted. For bacteriological analysis, BALF was plated onto several agar plates (Chapman's, MacConkey's, CNA and TSS agars) at 35°C for isolation of aerobic organisms. Cultures were examined for growth at 24 and 48 hours after incubation. Standard biochemical methods were used to identify cultured bacteria. The threshold used to define clinically relevant bacterial growth was 1.7 × 10^3^ colony‐forming units per milliliter of BALF.^33^ Bacterial susceptibility testing was performed according to standards of the Belgian and French Microbiology Societies using disk diffusion method. Finally, qPCR for *Bb* and *M*. *cynos* testing were performed by commercial veterinary diagnostic laboratories (TDDS Laboratories, University of Exeter, England and Laboratoire Scanelis, Colomiers, France).

### 2.2 Aerosolized delivery of gentamicin treatment

Immediately after *Bb* infection confirmation, aerosolized delivery of gentamicin was prescribed twice daily. Two protocols were successively used over the study period. Initially (from 2012 to the beginning of 2014), a dose 4 mg/kg was used twice daily with systematic dilution in saline to obtain *in toto* 5 mL of reconditioned solution nebulized until the cupule was empty (=protocol 1). The protocol was then modified since 2015 to a fixed concentration of gentamicin solution (gentamicin 5%) (2‐4 mL volume of undiluted gentamicin) nebulized over a minimum of 10 minutes independently of the dog size (=protocol 2). The time needed before the cupule was empty varied according to the type of nebulizer; if the cupule was empty before 10 minutes of nebulization, additional volume of undiluted gentamicin was added.

A pediatric nebulizer was used. Owners of dogs of less than 10 kg of body weight were instructed to place their dog in a feline transport cage adapted to the dog's size covered by a thick towel or blanket. Owners were also advised to wait a few minutes after the end of the nebulization before taking the dog out of the cage. For dogs of higher weight (>10 kg), the tube of the nebulizer was connected to a facial mask adapted to the size of the nose. Care was also taken to minimize owner's direct exposition to nebulized drug using distance from the dog during nebulization, limited space between mask and muzzle in >10 kg dogs or blanket over the transport cage in <10 kg dogs. For both protocols, nebulizations were initially prescribed twice daily for at least 3‐4 weeks and then pursued by additional 3 weeks‐increments until complete resolution of cough.

No cough suppressant was prescribed during the period study at both centers. Concomitant oral antibiotic treatment was initiated or pursued at the clinicians' discretion. At time of follow‐up, all owners were asked for clinical tolerance of nebulization at home.

Clinical cure was defined as the resolution of clinical signs (absence of cough, resolution of dyspnea, pyrexia, inappetence, and lethargy) without recurrence within 1 month after the interruption of nebulization. Follow‐up was carried out by both control visits and phone calls. After the last control visit, owners were instructed to inform the teaching hospital if cough re‐occurred thereafter, specifically within the first months after discontinuation of aerosols.

### 2.3 Statistical analysis

Statistical analyses were performed with a commercially available software (XLstat software). Data were expressed as median and range for continuous variables and as proportion for categorical variables.

Clinical response to aerosolized delivery of gentamicin was studied on 2 criteria: the proportion of dogs cured after 4 weeks and the median duration of treatment until clinical cure. Both criteria were studied depending on several factors including: age less than 6 months, breed (brachycephalic vs nonbrachycephalic dogs), acute presentation (cough for less than 2 weeks), presence or absence of co‐infection by *M*. *cynos* or other bacterial species, presence or absence of alveolar pattern on thoracic radiographs, history of previous steroid treatment, concomitant oral antimicrobial treatment during gentamicin protocol, use of cage vs facial mask, concomitant abnormal airways conformation at diagnosis, total cellularity of BALF (TCC less or more than 1000 cells/μL), severity of neutrophilic inflammation (less or more than 50% neutrophils in BALF) and finally, type of protocol (use of diluted or undiluted gentamicin).

Associations between proportion of dogs cured after 4 weeks and studied criteria were tested by Chi‐square test (with n > 5) and associations between median duration of treatment and studied criteria were tested by Mann‐Whitney test. Values of *P* ≤ .05 were considered significant.

## 3 RESULTS

Forty‐six dogs were included with 13 dogs treated with protocol 1 (13/13 diagnosed at the Veterinary Small Animal Teaching Hospitals of the University of Liege) and 33 dogs receiving protocol 2 (29 dogs diagnosed at the Veterinary Small Animal Teaching Hospitals of the University of Liege and 4 dogs diagnosed at the National School of Alfort). No dog was excluded. Owner compliance was deemed optimal regarding the gentamicin administration and clinical follow up was available for each dog.

Tables 1 and 2 present epidemiological, clinical and laboratory findings in dogs depending on the type of administered protocol. Median age of all dogs at diagnosis was 0.5 year (range 0.1‐7). Brachycephalic breeds, Cavalier King Charles spaniels, Yorkshire terriers and Chihuahua represented 58% (27/46) of the study population. The other represented breeds included: Spitz (n = 3), German Shepherd (n = 2), American Staffordshire terrier (n = 1), Alaskan Malamute (n = 1), Border Collie (n = 1), Basset Hound (n = 1), Chow Chow (n = 1), Jack Russel Terrier (n = 1), Labrador Retriever (n = 1), Maltese (n = 1), Münsterlander (n = 1), Pomeranian (n = 1), Setter (n = 1), Shar pei (n = 1), Shiba Inu (n = 1), and mixed‐breed dog (n = 1). Female and male dogs were equally represented (24/46 vs 22/46 respectively). Eight dogs were previously vaccinated; only parenteral vaccine was used for all these 8 dogs and was administered more than 2 months before diagnosis.

**TABLE 1:** Epidemio‐clinical findings of dogs treated with protocol 1 and protocol 2

	Protocol 1 (n = 13)	Protocol 2 (n = 33)
Less than 1 year old	10/13 (76%)	31/33 (93%)
Less than 6 month old	9/13 (69%)	22/33 (67%)
Median age (range)	0.5 year (0.2‐3.9)	0.5 year (0.1‐7)
Brachycephalic dogs	4/13 (30%)	10/33 (30%)
Dogs with body weight < 10 kg	7/13 (54%)	22/33 (67%)
Median body weight (range)	7.0 kg (2.0‐36.2)	5.4 kg (1.0‐34.2)
Previous vaccination	4/13 (30%)	4/33 (12%)
Dogs with cough of >2 weeks (chronic presentation)	12/13 (92%)	28/33 (85%)
Median duration of cough (range)	2 months (0.1‐24)	2 months (0.1‐12)
Dyspnea	6/13 (46%)	14/33 (42%)
Hyperthermia	3/13 (23%)	7/33 (21%)
Previous steroid treatment	0/13 (0%)	12/33 (36%)
Previous antibiotic treatment	Amoxicillin/clavulanic acid (n = 13) Marbofloxacin (n = 2) Enrofloxacin (n = 1) Doxycycline (n = 1) Cefovecin (n = 1)	Amoxicillin/clavulanic acid (n = 24) Doxycycline (n = 19) Marbofloxacin (n = 5) Enrofloxacin (n = 4) Pradofloxacin (n = 1) Cefalexin (n = 1) Spiramycin (n = 1) Metronidazole (n = 1) Azithromycin (n = 1)
Airways abnormalities other than brachycephalic problem Tracheal and bronchial collapse Epiglottic retroversion	0/13 (0%)	5/33 (15%) 5/33 1/33

*Note:* Brachycephalic dogs included Boxer and French and English Bulldog. Protocol 1: fixed dose of 4 mg/kg of gentamicin with dilution in saline; Protocol 2: fixed undiluted concentration of 5% of gentamicin solution.

**TABLE 2:** Laboratory findings and additional therapeutic data of dogs receiving protocol 1 and protocol 2

	Protocol 1 (n = 13)	Protocol 2 (n = 33)
Dogs with alveolar lesions on thoracic radiographs	8/13 (61%)	18/33 (54%)
Dogs with positive culture	7/13 (54%)	16/33 (48%)
Range of Ct (qPCR for Bb)	(19‐27.4)	(18‐25.9)
Median Ct (qPCR for Bb)	20	21
Dogs with co‐infection with other bacteria	2/13 (15%)	3/33 (9%)
Dogs with coinfection with *M*. *cynos*	7/12 (58%)	11/19 (57%)
Dogs with TCC of BALF >1000 cells/μL	7/13 (53%)	19/28 (67%)
Median TCC (range)	3280 (550‐39 000)	1700 (560‐52 900)
Dogs with % neutrophils on BALF >50%	9/13 (69%)	17/28 (60%)
Median % neutrophils (range)	83% (15‐90)	84% (30‐90)
Dogs with concomitant oral antimicrobial treatment	9/13 (69%)	13/33 (40%)
Use of transport cage for nebulization	7/13 (54%)	22/33 (67%)

*Note:* Protocol 1: fixed dose of 4 mg/kg of gentamicin with dilution in saline; Protocol 2: fixed undiluted concentration of 5% of gentamicin solution.

Abbreviations: BALF: bronchoalveolar lavage fluid; *Bb*: *Bordetella bronchiseptica*; *M*. *cynos*: *Mycoplasma cynos*; qPCR, quantitative polymerase chain reaction; TCC, total cell count of bronchoalveolar lavage.

All dogs had cough of 4 days to 2 years duration (median 2 months). Acute presentation (cough less than 2 weeks) was observed for 6 dogs, whereas for 36 dogs, cough was reported since adoption from kennels, pet shops or shelters. Twenty cases experienced moderate or severe dyspnea requiring hospitalization for oxygen treatment in the half of them. In 19 cases, mucous or purulent nasal discharge was observed associated with sneezing or reverse sneezing in 2 dogs. Ten dogs presented recurrent pyrexia of moderate severity (from 39.2 to 39.9°C) before diagnosis. All included dogs had been unsuccessfully treated with antimicrobial drugs including amoxicillin or amoxicillin/clavulanic acid (n = 37), doxycycline (n = 20), marbofloxacin or enrofloxacin (n = 13), cephalexin (n = 1), cefovecin (n = 1), spiramycin (n = 1), metronidazole (n = 1), and azithromycin (n = 1); clinical signs persisted for all dogs despite oral administration. Among these dogs, at the time of diagnosis, antibiotics had been stopped within the precedent 48 hours in 24 dogs and at least 1 week before BALF collection in the 22 remaining dogs. Twelve dogs had received anti‐inflammatory dosage of prednisolone before referral and corticosteroid treatment was interrupted after confirmation of diagnosis.

Radiographical alveolar pattern was observed in 25/46 dogs. In all dogs, total cell counts of BALF were elevated (median = 1780 cells/μL, range [550‐52 900] cells/μL). Neutrophilic airway inflammation was detected in 25/40 dogs for which differential counts of BALF were available (median = 83%, range [25%‐100%]). *Bb* infection was confirmed by bacterial culture in 23 dogs. For the others, qPCR on BALF was positive with a Ct level from 18 to 27.4 (high load). In 39 dogs, cytological examination of BALF revealed the presence of several coccobacilli adhered to cilia of epithelial cells. In 5 dogs, a non‐mycoplasmal co‐infection was detected by bacterial culture of BALF (*Staphylococcus intermedius* n = 3, *Klebsiella pneumoniae* n = 1, *Pseudomonas aeruginosa* n = 1). Among 31 dogs for which qPCR for *M*. *cynos* on BALF was available, 18 dogs were positive with a Ct level from 18.5 to 36.

After diagnosis, oral antibiotic treatment was concomitantly prescribed in 21 dogs. Amoxicillin/clavulanic acid, doxycycline or enrofloxacin was sometimes initiated or pursued at the clinicians' discretion, as dictated by the severity of clinical signs, radiographic findings (especially if alveolar pattern was present), and presence of bacterial co‐infections as demonstrated by conventional culture (choice based on antibiotic susceptibility pattern of the co‐agent).

Transport cage was used for gentamicin nebulization in 8 of the 13 dogs treated with protocol 1 and in 23 of the 33 dogs treated with protocol 2.

When dogs from both groups were compared (Tables 1 and 2), cases treated with protocol 2 were more frequently pretreated with steroids before inclusion (12/33, 36% vs 0/13, 0%, *P* = .01).

Table 3 presents the proportion of dogs with clinical cure at 3‐4 weeks and median duration of treatment for all dogs. Good clinical tolerance was reported by owners of all dogs. Clinical cure at 3‐4 weeks was more frequently observed in dogs treated with protocol 2 (19/33 [57%] vs 3/13 [23%] dogs, *P* = .03) and median duration of treatment was also significantly shorter (4 vs 6 weeks, *P* = .01). Moreover, proportion of dogs cured at 3‐4 weeks was significantly higher for dogs having TCC in BALF lower than 1000 cells/μL (11/15 [73%] vs 10/25 [40%], *P* = .04) but median duration of treatment was not different between dogs having TCC less than 1000 cells/μl and dogs having a higher TCC (4 weeks vs 6 weeks, *P* = .08).

**TABLE 3:** Number and proportion of dogs with clinical cure at 3‐4 weeks and median duration of treatment depending on studied criteria

	Cured at 3–4 weeks	*P* values (*Chi test*)	Median duration	*P* values (*Mann Whitney test*)
Less than 6‐month‐old n = 31	13/31 (*42%*)	.16	5	**.65**
More than 6‐month‐old n = 14	9/14 (*64%*)		4	
Brachycephalic dogs n = 13	5/13 (*38%*)	.37	5	**.79**
Non brachycephalic dogs n = 32	17/32 (*53%*)		4	
Dogs with cough <2 weeks n = 6	3/6 (*50%*)	1.00	4	**.68**
Dogs with cough >2 weeks n = 40	19/40 (*48%*)		5	
Co‐infection with other bacteria n = 4	3/4 (*75%*)	.27	4	**.71**
No co‐infection with other bacteria n = 41	19/41 (*46%*)		5	
Co‐infection with *M*. *cynos* n = 18	8/18 (*44%*)	.92	5	**.88**
No co‐infection with *M*. *cynos* n = 13	6/13 (*46%*)		5	
Radiographic alveolar lesions n = 25	11/25 (*44%*)	.46	5	**.44**
No radiographic alveolar lesions n = 20	11/20 (*55%*)		4	
Previous steroid treatment n = 12	6/12 (*50%*)	.93	4.5	**.87**
No previous steroid treatment n = 34	16/33 (*48%*)		5	
TCC of BALF >1000 cells/μL n = 25	10/25 (*40%*)	.04	6	**.08**
TCC of BALF <1000 cells/μL n = 15	11/15 (*73%*)		4	
% neutrophils on BALF >50% n = 25	12/25 (*48%*)	.46	5	**.53**
% neutrophils on BALF <50% n = 15	9/15 (*60%*)		4	
Concomitant oral antimicrobial treatment^a^ n = 21	9/21 (*43%*)	.36	5	**.49**
No concomitant oral antimicrobial treatment n = 23	13/23 (*57%*)		4	
Use of transport cage n = 31	15/31 (*48%*)	1.00	5	**.35**
Use of facial mask n = 15	7/15 (*47%*)		4.5	
Airways abnormalities n = 5	2/5 (*40%*)	.67	5	**.57**
No airways abnormalities n = 40	20/40 (*50%*)		4.5	
Protocol 1 n = 13	3/13 (*23%*)	.03	6, range 3–8 [interquartile: 4.7‐6]	**.01**
**Protocol 2 n = 33**	**19/33 (58%)**		**4 range 3–6 [interquartile**: **3‐4]**	

*Note:* Brachycephalic dogs included Boxer and French and English Bulldog. Airways abnormalities including tracheal or bronchial collapse and epiglottic retroversion. Protocol 1: fixed dose of 4 mg/kg of gentamicin with dilution in saline; Protocol 2: fixed undiluted concentration of 5% of gentamicin solution. Significance values are given in bold.

Abbreviations: BALF: bronchoalveolar lavage fluid; *M*. *cynos*: *Mycoplasma cynos*; qPCR, quantitative polymerase chain reaction; TCC, total cell count of bronchoalveolar lavage.

^a^ Amoxicillin/clavulanic acid, doxycycline, or enrofloxacin.

None of the other studied factors including age less than 6 months, breed, acute presentation (cough less than 2 weeks), presence or absence of any bacterial co‐infection (including *M*. *cynos*), radiographic alveolar pattern, history of previous corticosteroid treatment, amplitude of neutrophilic airway inflammation, concomitant oral antimicrobial treatment during gentamicin protocol, use of cage vs facial mask or concomitant abnormal airways conformation at diagnosis was associated with clinical cure at 3‐4 weeks or median treatment duration.

When only dogs treated with protocol 2 were considered (data not shown), similar results were obtained: association between proportion of dogs cured at 3‐4 weeks and TCC from BALF remained significative (9/9 vs 9/19, *P* = .006) and median duration of treatment was also shorter for dogs having TCC less than 1000 cells/μL in BALF (3 weeks vs 5 weeks, *P* = .02).

Authors considered a switch toward protocol 2 for 4 small‐sized dogs having poor clinical response with protocol 1. Those dogs included 1 Yorkshire Terrier dog of 1.3 kg, 1 Chihuahua dog of 1.0 kg and 2 French Bulldog dogs of 2.1 and 10.1 kg. In these 4 dogs, clinical signs persisted despite treatment of more than 8 weeks with protocol 1. After transition to protocol 2, cough resolved after 3‐6 weeks in all of them.

## 4 DISCUSSION

The present study documents clinical response to 2 protocols of aerosolized delivery of gentamicin in dogs diagnosed with *Bb* and previously treated unsuccessfully with oral antimicrobial treatment. Dogs treated with protocol 2 using undiluted solution nebulized during 10 minutes twice daily were more frequently cured at 3‐4 weeks than dogs receiving the protocol 1 using a fixed dose of 4 mg/kg. Median duration of treatment was also shorter with the protocol 2. Moreover, dogs having TCC from BALF less than 1000 cells/μL were more frequently cured at 3‐4 weeks than others. Lastly, 4 dogs achieved clinical cure only after switching from protocol 1 to protocol 2. Even though aerosolized delivery of gentamicin can appear time‐consuming, our study confirms its clinical benefit for *Bb* infection in dogs not responding to oral antimicrobial treatment.

Even if *Bordetella* is rarely antibiotic‐resistant in *in vitro* conditions,^25^ clinically‐refractory *Bordetella* infection is commonly observed.^8^ This can be related to the pathogenesis of this infection combining ciliostasis, biofilm formation,^19^, ^20^, ^21^, ^22^ local immunomodulation^22^, ^23^, ^24^ and the limited diffusion of oral or parenteral antimicrobial drugs at the apical cell surface in bronchi. Administration of antimicrobials by nebulization is thereby presumed to maximize drug delivery to the target site of infection with minimal or insignificant systemic absorption and thus limited or absent systemic adverse effects.

Inhaled aminoglycosides are classically used in human respiratory medicine in cases of *Pseudomonas* infection in patients with cystic fibrosis, cystic bronchiectasis or ventilator‐associated pneumonia^26^, ^27^, ^28^, ^36^, ^37^ with better clinical cure rates, less hospital stays and fewer days to reach complete recovery, compared to IV administration.^37^

In dogs with respiratory diseases, aerosolized delivery of gentamicin is sometimes used in practice despite of lack of literature on that topic. Indeed, there is only a single and old study reporting the use of aerosolized delivery of gentamicin for *Bb* infection in dogs^31^; doses and duration were not standardized and long‐term follow up was limited. In horses, use of nebulization with gentamicin has been documented. In healthy horses, aerosolized administration of undiluted solution of 5% gentamicin results in greater antimicrobial concentrations in bronchial fluid than after IV administration^39^ supporting once more this route of treatment. In foals, aerosolized administration of liposomal gentamicin results in higher intracellular drug concentration than with free gentamicin and emphasizes the interest of such galenic preparation for treating dogs in future trials.^40^

The present study illustrates the clinical benefit of nebulization of undiluted gentamicin (protocol 2) in the treatment of *Bb* infection in dogs. Variable dilution of gentamicin according to protocol 1 makes the total amount of inhaled gentamicin lower in small dogs compared to large dogs whereas in protocol 2, the use of undiluted solution is expected to allow inhalation of gentamicin in amounts proportional to the individual tidal volume, making it suitable in all dogs independently of their size.

We also observed that dogs having less than 1000 cells/μL in BALF were more frequently cured at 3‐4 weeks than dogs with more than 1000 cells/μL in lavage. Moreover, for dogs receiving undiluted solution, median duration of treatment was also significantly shorter with cellularity of lavage less than 1000 cells/μL. This observation seems relevant as a standardized BAL procedure was used. We can hypothesize that cellularity of BALF could be associated with disease severity, although validated clinical scoring was not available in our study population to confirm this hypothesis. High cellularity (more than 1000 cells/μL) could also suggest significant accumulation of suppurative secretions, which might limit optimal diffusion of gentamicin. Such finding poses TCC as an interesting clue of severity carrying prognostic value.

Interestingly, we failed to show a significant association of other studied criteria (young age, brachycephalic airway obstructive syndrome, acute presentation, previous steroid treatment, amplitude of neutrophilic airway inflammation, concurrent bacterial or mycoplasmal coinfections, concomitant oral antimicrobial treatment, use of transport cage rather than facial mask and presence of other airways abnormalities such as tracheobronchial collapse or epiglottic retroversion) on the clinical response or the needed duration of treatment. *M*. *cynos* has been identified as an emerging and potentially lethal pathogen in dogs such as in kennels or shelters.^4^, ^15^, ^16^, ^17^ Although *M*. *cynos* could be detected in asymptomatic shelter dogs,^6^, ^38^ the association of *M*. *cynos* with CIRD‐C is increasingly being recognized.^5^, ^7^, ^15^, ^16^, ^17^, ^18^ Nevertheless, the present study did not demonstrate an association between *M*. *cynos* co‐infection and the response to nebulized gentamicin treatment in dogs with *Bb* infection. Transport cages were used for the great majority of the recruited dogs. Small transport cages were considered adequate and well adapted for tiny dogs: animals feel comfortable and are able to breathe without stress and any restraint for a longer period of time. As those cages are quite small and relatively tight, they can be expected to be quickly saturated with the nebulized product. It is also a much more comfortable way to treat difficult or aggressive animals. Either with transport cage or facial mask, clinicians should anyway be concerned with limiting potential drug exposure of owners.

Furthermore, we did not observe any adverse effects related to systemic absorption of the drug, in accordance with previous studies assessing toxicity of aerosolized delivery of gentamicin. In an old study in healthy dogs receiving gentamicin aerosols up to 150 mg/dog, plasma concentration of gentamicin was undetectable.^34^ Moreover, no pulmonary histological lesions were found in dogs after maximal inhaled doses (up to 41 mg/kg over 1 hour) administered once daily during 2 weeks.^35^ Both last studies highlight the safety of aerosolized delivery of gentamicin in dogs.

Our retrospective study presents limitations. Because our study population is composed of referred and previously‐treated dogs, we propose to reserve such protocols for refractory *Bb* infection only. The interest of this route of treatment as first‐line option remains unevaluated and warrants further studies. Having 2 different delivery systems (cage vs mask nebulization) might represent a possible confounding factor. The use of cage nebulization makes the aerosolized delivery of gentamicin dependent on volume of the available air space available (sizes of used cages and animals). However, the proportion of dogs receiving nebulization in cages was not different between the 2 groups of treatment (Table 2), limiting a possible bias when evaluating the clinical response to both treatment protocols. Moreover, the use of a transport cage for nebulization was not statistically associated with clinical cure at 3‐4 weeks neither with median duration of treatment compared to dogs treated with facial mask. Nevertheless, the use of cage could expose the owner to unnecessary antimicrobials. In this respect, treatment with a better adapted nebulization device such as the new commercially‐available veterinary systems (eg, Flexineb C1 Portable System) should ideally be assessed and advised in a near future. Moreover, treatments were not randomized because of the retrospective design of the study and the comparison of different dosing protocols should be interpreted with great caution. Clinical response to protocol 2 could have been underestimated because dogs receiving protocol 2 were more frequently pretreated with steroids and were less frequently treated with concomitant oral antimicrobial drugs. Furthermore, several pediatric nebulizers of various brands were used over the study period, leading to potential variations in droplets size, power of the aerosolization and the deepness of the drug deposition. In addition, for ethical reason, our study did not include any group treated with saline only. Moreover, definitive cure was only defined based on clinical response, that is, absence of cough persisting at least 1 month after the interruption of aerosols. Even if clinical follow‐up was long, a second analysis of BALF collected at the end of treatment would have been more helpful to most adequately qualify definitive cure. However, such procedure requires anesthesia and most owners were reluctant to allow it while their pet appeared cured; thus, it was not considered in the study design for the inclusion. Additional studies including sequential BALF analysis after treatment is warranted to ascertain the efficacity of aerosolized administration of gentamicin. Lastly, although presence of brachycephalic obstructive airway syndrome would be expected to compromise optimal aerosolized drug delivery to the lower airways (particles colliding into upper airway mucosa due narrowed airways), we failed to observe a statistical difference in clinical response between brachycephalic and nonbrachycephalic dogs. We cannot rule out that this negative finding was related to the small size of the population in the present study (underpowered study).

In conclusion, while being more time‐consuming than oral antimicrobial treatment, treatment using nebulization of gentamicin in dogs with *Bb* infection seems to be well‐accepted. Twice daily nebulization using undiluted gentamicin solution is associated with a high proportion of dogs cured after 3‐4 weeks and with a shorter duration of treatment compared with the use of diluted gentamicin solution. Therefore, nebulized gentamicin can be advised for treating dogs with *Bb* infection that are poorly responsive to conventional antibiotic treatment and, at present, its use should currently be restricted to those cases. Based on the present positive clinical results with the protocol 2, complementary prospective randomized and placebo‐controlled studies with standardized therapeutic protocols using aerosols of undiluted gentamicin and commercially‐available veterinary systems (tight mask and flexible tube systems supplied with filters) are thus required.

## CONFLICT OF INTEREST DECLARATION

Authors declare no conflict of interest.

## OFF‐LABEL ANTIMICROBIAL DECLARATION

Nebulized gentamicin.

## INSTITUTIONAL ANIMAL CARE AND USE COMMITTEE (IACUC) OR OTHER APPROVAL DECLARATION

Authors declare no IACUC or other approval was needed.

## HUMAN ETHICS APPROVAL DECLARATION

Authors declare human ethics approval was not needed for this study.

## References

[1] Bemis DA , Greisen HA , Appel MJ . Pathogenesis of canine bordetellosis. J Infect Dis. 1977;135:753‐762. 404367 10.1093/infdis/135.5.753

[2] Bemis DA . *Bordetella* and *Mycoplasma* respiratory infections in dogs and cats. Vet Clin North Am Small Anim Pract. 1992;22:1173‐1186. 1523788 10.1016/s0195-5616(92)50308-4

[3] Keil DJ , Fenwick B . Role of *Bordetella bronchiseptica* in infectious tracheobronchitis in dogs. J Am Vet Med Assoc. 1998;212:200‐207. 9448823

[4] Priestnall SL , Mitchell JA , Walker CA , et al. New and emerging pathogens in canine infectious respiratory disease. Vet Pathol. 1992;51:492‐504. 10.1177/0300985813511130 24232191

[5] Schulz BS , Kurz S , Weber K . Detection of respiratory viruses and *Bordetella bronchiseptica* in dogs with acute respiratory tract infections. Vet J. 2014;201:365‐369. 24980809 10.1016/j.tvjl.2014.04.019 PMC7110455

[6] Lavan R , Knesl O . Prevalence of canine infectious respiratory pathogens in asymptomatic dogs presented at US animal shelters. J Small Anim Pract. 2015;56:572‐576. 26199194 10.1111/jsap.12389 PMC7166506

[7] Decaro N , Mari V , Larocca V , et al. Molecular surveillance of traditional and emerging pathogens associated with canine infectious respiratory disease. Vet Microbiol. 2016;192:21‐25. 27527760 10.1016/j.vetmic.2016.06.009 PMC7131703

[8] Canonne AM , Billen F , Tual C , et al. Quantitative PCR and cytology of bronchoalveolar lavage fluid in dogs with *Bordetella bronchiseptica* infection. J Vet Intern Med. 2016;30:1204‐1209. 27461723 10.1111/jvim.14366 PMC5108481

[9] Wöhrer D , Sperger J , Bagrinovschi K , et al. Age‐related presence of selected viral and bacterial pathogens in paraffin‐embedded lung samples of dogs with pneumonia. Acta Vet Hung. 2016;64(1):103‐115. 26919147 10.1556/004.2016.011

[10] Taha‐Abdelaziz K , Bassel LL , Harness ML , Clark ME , Register KB , Caswell JL . Cilia‐associated bacteria in fatal *Bordetella bronchiseptica* pneumonia of dogs and cats. J Vet Diagn Invest. 2016;28:369‐376. 27178716 10.1177/1040638716646806

[11] Radhakrishnan A , Drobatz KJ , Culp WT , et al. Community‐acquired infectious pneumonia in puppies: 65 cases (1993‐2002). J Am Vet Med Assoc. 2007;230:1493‐1497. 17504040 10.2460/javma.230.10.1493

[12] Dworkin MS , Sullivan PS , Buski SE , et al. *Bordetella bronchiseptica* infection in human immunodeficiency virus‐infected patients. Clin Infect Dis. 1999;28:1095‐1099. 10452641 10.1086/514761

[13] Ner Z , Ross LA , Horn MV , et al. *Bordetella bronchiseptica* infection in pediatric lung transplant recipients. Pediatr Transplant. 2003;7:413‐417. 14738306 10.1034/j.1399-3046.2003.00074.x

[14] Yacoub AT , Katayama M , Tran J , Zadikany R , Kandula M , Greene JN . *Bordetella bronchiseptica* in the immunosuppressed population ‐ a case series and review. Mediterr J Hematol Infect Dis. 2014;6:e2014031. 24804004 10.4084/MJHID.2014.031 PMC4010603

[15] Rycroft A , Tsounakou E , Chalker V . Serological evidence of *Mycoplasma cynos* infection in canine infectious respiratory disease. Vet Microbiol. 2007;120:358‐362. 17184934 10.1016/j.vetmic.2006.11.011 PMC7127756

[16] Zeugswetter F , Weissenböck H , Shibly S , et al. Lethal bronchopneumonia caused by *Mycoplasma cynos* in a litter of golden retriever puppies. Vet Rec. 2007;161:626‐627. 17982143 10.1136/vr.161.18.626

[17] Mannering S , Mcauliffe L , Lawes J , et al. Strain typing of *Mycoplasma cynos* isolates from dogs with respiratory disease. Vet Microbiol. 2009;135:292‐296. 18977617 10.1016/j.vetmic.2008.09.058 PMC7126931

[18] Sowman HR , Cave NJ , Dunowska M . A survey of canine respiratory pathogens in New Zealand dogs. N Z Vet J. 2018;66:236‐242. 29924957 10.1080/00480169.2018.1490214

[19] Anderton TL , Maskell DJ , Preston A . Ciliostasis is a key early event during colonization of canine tracheal tissue by *Bordetella bronchiseptica* . Microbiology. 2004;150:2843‐2855. 15347744 10.1099/mic.0.27283-0

[20] Buboltz AM , Nicholson TL , Weyrich LS , Harvill ET . Role of the type III secretion system in a hypervirulent lineage of *Bordetella bronchiseptica* . Infect Immun. 2009;77:3969‐3977. 19596779 10.1128/IAI.01362-08 PMC2738013

[21] Cattelan N , Dubey P , Arnal L , Yantorno OM , Deora R . *Bordetella* biofilms: a lifestyle leading to persistent infections. Pathog Dis Feb. 2016;74:ftv108. 10.1093/femspd/ftv108 PMC4830220 26586694

[22] Pilione MR , Harvill ET . The *Bordetella Bronchiseptica* type III secretion system inhibits gamma interferon production that is required for efficient antibody‐mediated bacterial clearance. Infect Immun. 2006;74:1043‐1049. 16428751 10.1128/IAI.74.2.1043-1049.2006 PMC1360352

[23] Skinner JA , Pilione MR , Shen H , Harvill ET , Yuk MH . *Bordetella* type III secretion modulates dendritic cell migration resulting in immunosuppression and bacterial persistence. J Immunol. 2005;175:4647‐4652. 16177111 10.4049/jimmunol.175.7.4647

[24] Siciliano NA , Skinner JA , Yuk MH . *Bordetella bronchiseptica* modulates macrophage phenotype leading to the inhibition of CD4+ T cell proliferation and the initiation of a Th17 immune response. J Immunol. 2006;177:7131‐7138. 17082630 10.4049/jimmunol.177.10.7131

[25] Steinfeld A , Prenger‐Berninghoff E , Bauer N , Weiß R , Moritz A . Bacterial susceptibility testings of the lower airways of diseased dogs. Tierarztl Prax Ausg K Kleintiere Heimtiere. 2012;40:309‐317. 23076014

[26] Murray MP , Govan JR , Doherty CJ , et al. A randomized controlled trial of nebulized gentamicin in non‐cystic fibrosis bronchiectasis. Am J Respir Crit Care Med. 2011;183:491‐499. 20870753 10.1164/rccm.201005-0756OC

[27] Yang JW , Fan LC , Lu HW , Miao XY , Mao B , Xu JF . Efficacy and safety of long‐term inhaled antibiotic for patients with noncystic fibrosis bronchiectasis: a meta‐analysis. Clin Respir J. 2016;10:731‐739. 25620629 10.1111/crj.12278

[28] Soltaninejad F , Kheiri S , Habibian R , et al. Evaluation effects of nebulized gentamicin in exacerbation of chronic obstructive lung disease. J Res Med Sci. 2016;21:56. 27904601 10.4103/1735-1995.187278 PMC5122183

[29] Heyder J . Deposition of inhaled particles in the human respiratory tract and consequences for regional targeting in respiratory drug delivery. Proc Am Thorac Soc. 2004;1(4):315‐320. 16113452 10.1513/pats.200409-046TA

[30] Lipworth B , Manoharan A , Anderson W . Unlocking the quiet zone: the small airway asthma phenotype. Lancet Respir Med. 2014;2:497‐506. 24899370 10.1016/S2213-2600(14)70103-1

[31] Bemis DA , Appel MJ . Aerosol Nolvasan treatment of *Bordetella bronchiseptica* in dogs. Vet Med Small Anim Clin. 1977;72:53‐55. 584098

[32] Dehard S , Bernaerts F , Peeters D , et al. Comparison of bronchoalveolar lavage cytospins and smears in dogs and cats. J Am Anim Hosp Assoc. 2008;44:285‐294. 18981193 10.5326/0440285

[33] Peeters D , McKiernan BC , Weisiger RM , et al. Quantitative bacterial cultures and cytological examination of bronchoalveolar lavage specimens in dogs. J Vet Intern Med. 2000;14:534‐541. 11012118 10.1892/0891-6640(2000)014<0534:qbcace>2.3.co;2

[34] Riviere JE , Silver GR , Coppoc GL , Richardson RC . Gentamicin aerosol therapy in 18 dogs: failure to induce detectable serum concentrations of the drug. J Am Vet Med Assoc. 1981;179:166‐168. 7263469

[35] Conway H , Dix KJ , McDonald JD , et al. Comparison of inhalation toxicity studies of gentamicin in rats and dogs. Inhal Toxicol. 2013;25:714‐724. 24255949 10.3109/08958378.2013.843043

[36] Boisson M , Mimoz O , Hadzic M , et al. Pharmacokinetics of intravenous and nebulized gentamicin in critically ill patients. J Antimicrob Chemother. 2018;73:2830‐2837. 29947799 10.1093/jac/dky239

[37] Hassan NA , Awdallah FF , Abbassi MM , Sabry NA . Nebulized versus IV Amikacin as adjunctive antibiotic for hospital and ventilator‐acquired pneumonia Postcardiac surgeries: a randomized controlled trial. Crit Care Med. 2018;46:45‐52. 28857848 10.1097/CCM.0000000000002695

[38] Joffe DJ , Lelewski R , Weese J , et al. Factors associated with development of canine infectious respiratory disease complex (CIRDC) in dogs in 5 Canadian small animal clinics. Can Vet J. 2016;57:46‐51. 26740697 PMC4677608

[39] McKenzie HC , Murray MJ . Concentrations of gentamicin in serum and bronchial lavage fluid after intravenous and aerosol administration of gentamicin to horses. Am J Vet Res. 2000;61:1185‐1190. 11039545 10.2460/ajvr.2000.61.1185

[40] Burton AJ , Giguère S , Arnold RD . Pharmacokinetics, pulmonary disposition and tolerability of liposomal gentamicin and free gentamicin in foals. Equine Vet J. 2015;47:467‐472. 24943347 10.1111/evj.12309

